# Effect of exclusive breastfeeding and other infant and young child feeding practices on childhood morbidity outcomes: associations for infants 0–6 months in 5 South Asian countries using Demographic and Health Survey data

**DOI:** 10.1186/s13006-024-00644-x

**Published:** 2024-05-16

**Authors:** Saldana Hossain, Seema Mihrshahi

**Affiliations:** Department of Health Sciences, Faculty of Medicine, Health and Human Sciences, Macquarie University, Sydney, NSW 2109 Australia

**Keywords:** Exclusive breastfeeding, South Asia, Diarrhoea, Acute respiratory infection, Fever

## Abstract

**Background:**

Despite growing evidence of the impacts of exclusively breastfeeding infants during the first 6 months of life on preventing childhood infections and ensuring optimal health, only a small number of studies have quantified this association in South Asia.

**Methods:**

We analyzed data from the Demographic and Health Surveys in Afghanistan (2015; *n* = 3462), Bangladesh (2017–2018; *n* = 1084), India (2019–2021; *n* = 26,101), Nepal (2022; *n* = 581), and Pakistan (2017–2018; *n* = 1,306), including babies aged 0–6 months. Multivariate logistic regression models were used to determine the association between exclusive breastfeeding in the last 24 h and diarrhoea, acute respiratory infections, and fever in the two weeks before the survey. We also examined the association between other infant and young feeding indicators and these outcomes.

**Results:**

Infants who were exclusive breastfed had decreased odds of diarrhoea in Afghanistan (AOR: 0.49, 95% CI 0.35, 0.70), India (AOR: 0.80, 95% CI 0.70, 0.91), and Nepal (AOR: 0.42, 95% CI 0.20, 0.89). Compared with infants who were not exclusive breastfed, infants who were exclusively breastfed were less likely to have fever in Afghanistan (AOR: 0.36, 95% CI 0.26, 0.50) and India (AOR: 0.75, 95% CI 0.67, 0.84). Exclusive breastfeeding was associated with lower odds of acute respiratory infections in Afghanistan (AOR: 0.57, 95% CI 0.39, 0.83). Early initiation of breastfeeding was protective against diarrhoea in India. Bottle feeding was a risk factor for diarrhoea in India and for fever in Afghanistan and India. Bottle feeding was also a risk factor for acute respiratory infection in Afghanistan and India.

**Conclusions:**

Not exclusive breastfeeding is a risk factor for diarrhoea, acute respiratory infections, and fever in some South Asian countries. These findings could have substantial implications for global and national efforts to increase exclusive breastfeeding rates. More support, advocacy, and action are required to boost breastfeeding rates as a crucial public health measure.

**Supplementary Information:**

The online version contains supplementary material available at 10.1186/s13006-024-00644-x.

## Background

According to United Nations Children’s Fund (UNICEF), about 9% of all childhood deaths under the age of five globally in 2019 were due to diarrhoea, making it one of the major causes of mortality in children [1]. More than 12 million children under the age of five are admitted to hospitals every year as a result of acute respiratory tract infections (ARI) [2]. Among them, acute lower respiratory infections (ALRIs) which includes pneumonia and bronchiolitis have emerged as one of the primary reasons for pediatric hospital admissions and in-hospital mortality of young children, particularly in low-income nations [3].

In recent years, an increasing number of research articles have been published on the benefits of optimal breastfeeding practices for the mother-infant pair [4–6]. Breastmilk has many anti-inflammatory and immunological properties that safeguard babies against a range of diseases [4, 7]. Exclusive breastfeeding (EBF) offers several known short- and long-term advantages, especially in lowering childhood morbidity and death from respiratory and diarrhoeal illnesses [5, 6]. Findings from a cohort study [8] conducted in eight different low-income countries of 1731 babies found that EBF was protective against the incidence of diarrhoea and ALRI. Breastfeeding has also shown to contribute to increased intelligence scores and academic performance, lower risk of diabetes and obesity in the long term, and a decreased risk of breast and ovarian cancer in mothers [9]. EBF for the first six months of a baby's life is recommended by the World Health Organization (WHO) in its policy guidelines in order to ensure the baby's adequate growth, development, and health [10]. At six months, the WHO recommends introducing nutritionally rich, safe, and appropriate complementary foods while continuing to breastfeed for at least two years [10].

Approximately 18% of acute respiratory deaths, 30% of diarrhoeal deaths among children under the age of 5, and 45% of newborn deaths from infections are attributed to inadequate breastfeeding practices [11]. Also, around 1.4 million under-five deaths and 10% of the disease burden in children are attributed to sub-optimal breastfeeding, particularly non-exclusive breastfeeding during the first six months of life [12]. According to evidence on the efficacy of interventions, attaining universal coverage of appropriate breastfeeding may avoid 13% of deaths in children under five years old worldwide [12]. Despite the advantages of optimum breastfeeding, limited improvement was seen in EBF rates between and across countries/regions and only 44% of babies worldwide aged 0 to 6 months were EBF from 2015 to 2020 [13]. Worldwide, breastfeeding rates are still below what is necessary to protect children's health. In 2012, the World Health Assembly set a goal of achieving a global EBF rate of at least 50% by the year 2025 [9]. In order to achieve this target, countries need to strengthen their efforts to increase breastfeeding rates.

Sub-Saharan Africa and South Asia had the highest neonatal mortality rates estimated to be 27 and 24 deaths per 1,000 live births, respectively, in 2020 [14]. UNICEF has also reported that babies born in South Asia had a nine-fold higher mortality rate than those born in high-income countries [14]. South Asia and sub-Saharan Africa account for the majority of deaths from diarrhoea among children under the age of five [15]. Almost 90% of diarrhoeal and pneumonia mortality occurred in South Asia and sub-Saharan Africa [15, 16]. Due to inadequate breastfeeding practices, insufficient access to immunization, unsafe water and unimproved sanitation facilities, and lack of access to treatment, the effect of diarrhoea-associated morbidity and mortality on infant survival is greatest among children from low-and middle-income countries (LMICs) [16, 17].

Rates of EBF have increased slightly around the world, with South Asia being the main driver of this change [18]. Between 2000 and 2015, this region's rate of EBF for babies aged 0–5 months rose from 47 to 64 percent, an increase of 17 percentage points [18]. Despite improvements on feeding practices in South Asia, there is substantial variation in breastfeeding practices across and within countries [19]. Breastfeeding practices vary across Bangladesh, India, Afghanistan, Pakistan, and Nepal, with breastfeeding initiation rates ranging from 19.6% in Pakistan to 59.8% in Bangladesh and EBF rates for infants aged 0–5 months remain suboptimal, ranging from 43.3% in Afghanistan to 63.7% in India [20–24]. Bottle feeding is prevalent in children under 2, with rates as high as 48% in Pakistan [23]. Since 2000, India, Pakistan and Bangladesh, among 15 consistently affected countries, have borne a significant share of global pneumonia and diarrhoea deaths in children under 5, with over two-thirds of the mortality burden concentrated in these 15 countries, highlighting the urgent need for interventions in these regions [25]. Socio-cultural factors such as maternal education, husband's education, working status, wealth, and family structure influence breastfeeding initiation practices, with variations observed across Bangladesh, India, Nepal, and Pakistan [26]. Evidence indicates that infant characteristics (small infant size), healthcare factors (poor use of antenatal services, home delivery, and caesarean delivery), and sociocultural norms such as prelacteal feeding are all barriers to EBF [19, 27, 28]. A better understanding of this variability is needed to guide policies and interventions to achieve the global aim of the World Health Assembly on EBF by 2025 [9, 19].

Several recent studies have explored the relationship between infant and young child feeding practices and infant morbidity outcomes using data from the Demographic and Health Surveys (DHS). For instance, a study by Ogbo et al. [29] pooled DHS data from multiple sub-Saharan African countries. While these studies have provided valuable insights, there is still a need to analyze these relationships in diverse settings and populations. Many studies have focused on individual countries or regions, limiting the generalizability of their findings. There is still a critical gap in the literature in South Asian countries with high diarrhoea and ARI morbidity using nationwide population-based data. As these populations have one of the highest disease burden in the region, it is crucial to examine these countries as interventions would have the greatest effect here [29]. Furthermore, comparison of countries has the benefit of illustrating how health status and characteristics differs among nations in a region to develop locally acceptable and context-specific infant feeding-related programmes and policies [27, 29]. The present study seeks to build upon existing literature by providing current evidence using the latest DHS data for Afghanistan (2015), Bangladesh (2017–2018), India (2019–2021), Nepal (2022), and Pakistan (2017–2018) [20–24]. We assessed the relationship between EBF and diarrhoea, ARI, and fever. In addition, we explored the association between other infant and young child feeding (IYCF) indicators and these health outcomes.

## Methods

### Data source, data collection, and study population

We did a secondary analysis using cross-sectional data from the most recent Demographic and Health Surveys (DHS) for the countries: Afghanistan (2015), Bangladesh (2017–2018), India (2019–2021), Nepal (2022), and Pakistan (2017–2018). The survey collects data on health of mothers and their children, nutritional outcomes, fertility, HIV/AIDS, immunization, and family planning (The DHS program). Within all countries, DHS follow the same sampling procedures to generate nationally and sub-nationally (region-wide/statewide) representative data [30]. A stratified two-stage random sampling design was used. In stage one, each nation was categorized into regions, which could be either geographical regions or political areas like provinces or states and named north, south, east, and west or political [30]. Populations in these subnational regions were divided into rural and urban areas of residence. A random selection of enumeration regions from the latest population census were chosen from within these stratified zones. The likelihood of each main sampling unit/cluster being chosen was set such that it corresponded to the percentage that each cluster's population contributed to the overall population [30]. In stage two, every house within the cluster were listed, and through equal-probability systematic selection, an average of 25 households in a cluster were randomly chosen for an interview. Further details on tools of data collection, DHS sampling techniques and house listing methods is published in country-specific DHS reports [30, 31].

The survey data were comparable across countries and survey years due to the use of standardized measurement techniques and survey instruments [31]. Women aged 15–49 years were given a questionnaire to obtain data on household demographics, maternal and child health. We used data from the youngest living infants aged less than 7 months who were living with their mother aged 15–49 years. Participants with missing data on variables such as exclusive breastfeeding, diarrhoea, acute respiratory infection, and fever were excluded.

### Outcome and exposure variables

The outcomes of interest were morbidity status of babies, which were measured based on any of the three morbidities: diarrhoea, ARI, or fever. Each of these variables were coded to indicate whether the outcome was present or not (“Yes” vs “No”). The IYCF indicators were the study's explanatory factors, measured according to the WHO’s definitions for assessing IYCF practices [32]. The primary exposure variable was exclusive breastfeeding. The specific definitions of all variables are described in Table 1.

**Table 1:** Measurement of exposure and outcomes variables

Variable	Definition
Exposure variable	
EBF	The percentage of babies who were fed breast milk as the only source of nutrition but allowed drops or syrups of vitamins, medicines, and oral rehydration solution To determine EBF status of babies from birth to 6 months of age, mothers were asked (i) whether the baby was still being breastfed; (ii) the duration of breastfeeding; and (iii) whether they had given the baby any other milk, liquids, solids, powdered milk, soft foods, or water in the 24 h preceding the interview
Early initiation of breastfeeding	The percentage of babies within 0–6 months of age who commenced breastfeeding within 1 h of birth
Bottle feeding	The percentage of babies 0–6 months of age who were fed any liquid (including breast milk) or semi-solid food from a bottle with nipple/teat during the last 24 h
Outcome variable	
Diarrhoea	Defined as the passage of three or more loose or liquid stools per day. This was based on maternal recall of symptoms of the infant in the last 2 weeks preceding the survey
ARI	Mothers were asked if the infant had a cough accompanied by short, rapid breasting that was chest-related and/or have difficulty breathing which was chest-related during the 2 weeks before the survey
Fever	Mothers were asked if the infant had been ill with a fever at any time during the last 2 weeks

### Covariates

Based on existing literature, we identified potential confounders and other determinants of breastfeeding practices in South Asia [27, 33–36]. Potential confounding factors were broadly classified into individual-level characteristics (infant age, mother’s age, gender), socio-economic characteristics (maternal education, working status of mother), health service factors (frequency of antenatal care (ANC) visits, place of delivery), and household-level characteristics (household wealth index, place of residence, number of listed household members, source of drinking water, toilet facility, cooking fuel).

Type of cooking fuel was used in the regression models for ARI, as some papers in South Asia reported the use of solid fuel as a risk factor for ARI [37, 38]. Solid fuels included charcoal, animal dung, wood, straw/grass/shrubs, agricultural crop, and kerosene, whereas clean fuels included natural gas, electricity, biogas, and liquefied petroleum gas (LPG). The type of toilet facility and the source of drinking water were classified as “improved” or “unimproved” in accordance with the WHO and UNICEF Joint Monitoring Programme (JMP) for Water and Sanitation [39]. Type of toilet facility was classified as ‘improved’ (included flush toilets piped to the sewer system, pit latrine or septic tank, flush to an unknown location, ventilated improved pit (VIP) latrine, composting toilet, pit latrine with slab). ‘Unimproved’ type of facility included flush not piped to sewer, hanging toilet/latrine, pit latrine without slab/open pit, bucket toilet and no facility/field/bush. `Improved' sources of water were defined as a piped water into dwelling/yard/plot/neighbor, public tap or standpipe, tube-well or borehole, protected spring and well, rainwater, cart with small tank, tanker truck, bottled water, filtration plant, while households that utilized unprotected spring and well, and surface water were classified as ‘unimproved’.

### Statistical analysis

All analyses were carried out using STATA V.17.0 (StataCorp). Descriptive statistics of the study sample were computed as frequencies with weighted percentages for the explanatory, outcome, and control variables. Bivariate analyses using Pearson’s chi-squared test was used to compare the prevalence of EBF among the levels of each covariate. All sample sizes and proportions were based on sampling weights to account for the survey design. Given the dichotomous nature of the outcome variables, multiple binary logistic regression models were fitted to examine the association between each exposure of interest, (1) EBF (2) early initiation of breastfeeding and (3) bottle feeding with diarrhoea, ARI, and fever (separate models for each outcome). All variables were entered into the initial model and variables with a *P* value of < 0.10 or considered to be conceptually relevant potential confounders regardless of *P* value were retained in the final multivariable logistic regression model. Unadjusted and adjusted odds ratios (AOR) with 95% confidence intervals (CIs) were obtained from the regression models with significance at the 5% level (*p* < 0.05). Full regression models with unadjusted ratios are detailed in Additional file 1. Variance inflation factor (VIF) was utilized to assess multicollinearity between the variables prior to running the models. The VIF test indicated a lack of high multicollinearity among the variables (VIF less than five). Multiple logistic regression models were fitted to the data separately for each country. We applied the ‘svy’ command in all our analyses to allow for the cluster sampling design of DHS surveys. The forest plot of AORs and 95% CIs for each country separately as well as the unweighted overall estimates for all five countries combined were created using STATA’s “metan” function.

## Results

### Characteristics of the included surveys

The characteristics of the women and children included in the surveys are summarized in Table 2. The total sample size ranged from 603 in Nepal to 26,219 in India. The mean age for women in the survey ranged from 24 to 27 years old. Bangladesh had a larger number of young mothers (23.3%) who were between 15- to 19-year-old compared to other countries. Meanwhile, Afghanistan had a larger number of mothers (5.3%) who were between 40 to 49 years old compared to other countries. Afghanistan had the greatest number of women (80.9%) who did not attain any schooling. Across all surveys, the majority of the women (more than 69%) did not work. Nepal had the highest number of women living in an urban area (68.4%), while most women in the other countries resided in rural areas. Approximately half of the women in Afghanistan (55.9%) and Pakistan (48.4%) had more than 9 people living in the house. Most women (approximately 81.3%) resided in households that used improved sources of drinking water in South Asia. Afghanistan had the highest proportion of unimproved drinking water sources (25.1%) and unimproved sanitation facility (70%) relative to the other countries. More than half of women lived in houses with improved drinking water source and toilet facility in Bangladesh, India, Pakistan, and Nepal. Also, Nepal (3.4%) and India (5.2%) had the lowest proportion of mothers who did not have any antenatal care, whereas Afghanistan had the highest (34.2%). More than 50% of women in these South Asian countries delivered their babies in a health facility. India had the greatest number of health facility deliveries (90.6%), while Bangladesh had the lowest (51.3%).

**Table 2:** Selected infant, maternal, household, and IYCF characteristics among five countries

	**Afghanistan (2015)** (*n* = 3573) **n (%)**	**Bangladesh (2017/18)** (*n* = 1087) **n (%)**	**India (2019/21)** (*n* = 26219) **n (%)**	**Nepal (2022)** (*n* = 603) **n (%)**	**Pakistan (2017/18)** (*n* = 1312) **n (%)**
***Infant characteristics***					
***Infant age (months)***					
0–2	1324 (37.1)	521 (47.9)	11,034 (42.1)	257 (42.6)	565 (43.1)
3–4	1253 (35.1)	297 (27.3)	7637 (29.1)	164 (27.2)	379 (28.9)
5–6	996 (27.9)	270 (24.8)	7547 (28.8)	182 (30.2)	368 (28.0)
***Gender***					
Female	1701 (47.6)	514 (47.3)	12,769 (48.7)	294 (48.8)	707 (53.9)
Male	1872 (52.4)	573 (52.8)	13,449 (51.3)	309 (51.2)	605 (46.1)
***Mother’s characteristics***					
***Mother’s age in years***					
15–19	287 (8.0)	253 (23.3)	2046 (7.8)	97 (16.0)	103 (7.8)
20–29	2191 (61.3)	641 (59.0)	19,790 (75.5)	398 (66.1)	769 (58.6)
30–39	904 (25.3)	184 (17.0)	4204 (16.0)	104 (17.3)	402 (30.7)
40–49	190 (5.3)	9 (0.8)	178 (0.7)	4 (0.6)	38 (2.9)
Mean	27.0	24.1	25.1	24.6	27.2
***Maternal education***					
No education	2890 (80.9)	69 (6.4)	4780 (18.2)	95 (15.7)	614 (46.8)
Primary	325 (9.1)	305 (28.1)	2935 (11.2)	203 (33.6)	223 (17.0)
Secondary	291 (8.2)	523 (48.1)	13,706 (52.3)	281 (46.6)	276 (21.0)
Higher	67 (1.9)	190 (17.5)	4798 (18.3)	25 (4.1)	200 (15.2)
***Mother’s employment***					
Working	363 (10.2)	307 (28.2)	371 (9.2)	183 (30.4)	123 (9.4)
Not working	3205 (89.7)	780 (71.8)	3646 (90.8)	420 (69.6)	1189 (90.6)
***Socioeconomic characteristics***					
***Type of residence***					
Rural	2698 (75.5)	792 (72.8)	19,768 (75.4)	191 (31.6)	888 (67.6)
Urban	875 (24.5)	295 (27.2)	6450 (24.6)	412 (68.4)	425 (32.4)
***Household size (members)***					
1–4	284 (8.0)	258 (23.7)	5176 (19.7)	132 (21.9)	144 (11.0)
5–8	1293 (36.2)	646 (59.4)	15,926 (60.8)	360 (59.7)	533 (40.6)
9 +	1996 (55.9)	183 (16.9)	5116 (19.5)	111 (18.5)	636 (48.4)
***Wealth index***					
Poorest	621 (17.4)	222 (20.4)	6292 (24.0)	119 (19.7)	300 (22.8)
Poorer	743 (20.8)	215 (19.8)	5789 (22.1)	129 (21.3)	226 (17.2)
Middle	818 (22.9)	226 (20.8)	5145 (19.6)	119 (19.8)	285 (21.7)
Richer	701 (19.6)	220 (20.2)	4904 (18.7)	122 (20.3)	257 (19.6)
Richest	690 (19.3)	204 (18.7)	4090 (15.6)	114 (18.9)	245 (18.7)
***Source of drinking water***					
Improved	2638 (73.9)	849 (78.1)	22,124 (84.4)	484 (80.3)	1180 (89.9)
Unimproved^a^	896 (25.1)	12 (1.1)	990 (3.8)	6 (1.0)	54 (4.1)
***Type of toilet facility***					
Improved	1033 (28.9)	562 (51.7)	16,961 (64.7)	445 (73.7)	976 (74.3)
Unimproved^b^	2501 (70.0)	299 (27.5)	6153 (23.5)	46 (7.6)	258 (19.6)
***Type of cooking fuel***					
Clean	1112 (31.2)	191 (17.6)	11,069 (42.2)	195 (32.3)	527 (40.1)
Solid	2408 (67.5)	666 (61.4)	12,017 (45.8)	296 (49.0)	706 (53.8)
***Health service use***					
***Number of ANC visits***					
None	1219 (34.2)	98 (9.0)	1363 (5.2)	20 (3.4)	127 (9.7)
1–3	1617 (45.3)	488 (44.9)	9351 (35.7)	88.8 (14.7)	470 (35.8)
4 or more	690 (19.4)	501 (46.1)	15,196 (58.0)	494 (81.9)	715 (54.5)
***Place of delivery***					
Health facility	2063 (57.8)	557 (51.3)	23,760 (90.6)	497 (82.5)	930 (71.0)
Home	1492 (41.8)	524 (48.2)^c^	2407 (9.2)	94 (15.6)	378 (28.8)
***IYCF indicators***					
***Exclusive breastfeeding***					
Yes	1405 (39.3)	635 (58.5)	15,326 (58.5)	306 (50.8)	556 (42.4)
No	2100 (58.8)	451 (41.5)	10,836 (41.3)	280 (46.4)	753 (57.4)
***Early initiation of breastfeeding***					
Yes	1604 (44.9)	614 (56.5)	10,748 (41.0)	329 (54.5)	272 (20.7)
No	1968 (55.1)	473 (43.5)	15,470 (59.0)	274 (45.5)	1040 (79.3)
***Bottle feeding***					
Yes	704 (19.7)	155 (14.2)	3433 (13.1)	107 (17.8)	507 (38.6)
No	2844 (79.6)	930 (85.5)	22,780 (86.9)	495 (82.2)	805 (61.3)
***Diarrhoea***					
No	2767 (77.6)	1054 (97.0)	23,979 (91.5)	523 (86.7)	1013 (77.2)
Yes	786 (22.0)	33 (3.0)	2219 (8.5)	78 (13.0)	299 (22.8)
***Acute respiratory infection***					
No	3184 (89.1)	1047 (96.3)	25,351 (96.7)	589 (97.7)	1128 (86.0)
Yes	371 (10.4)	38 (3.5)	819 (3.1)	11 (1.8)	181 (13.8)
***Fever***					
No	2685 (75.3)	758 (69.7)	23,266 (88.7)	494 (81.9)	851 (64.9)
Yes	868 (24.3)	329 (30.3)	2940 (11.2)	109 (18.1)	460 (35.1)

^a^Unimproved drinking water sources includes unprotected spring and well, and surface water, and other sources

^b^Unimproved toilet facilities include flush not piped to sewer, hanging toilet/latrine, pit latrine without slab/open pit, bucket toilet and no facility/field/bush and other types

^c^Delivery huts in Pakistan DHS are categorized as missing as they not considered home or facility births

**Fig. 1:**
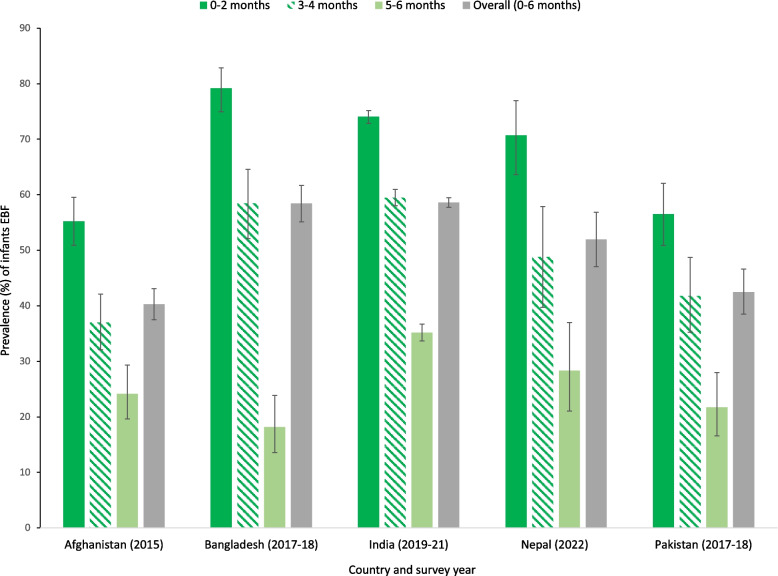
Prevalence of babies aged 0–6 months exclusively breastfed by age group in South Asian countries. Error bars represent 95% confidence intervals (CI). EBF: Exclusive breastfeeding

As expected, the percentage of babies who were EBF declined with infant age in all countries (Fig. 1) and the prevalence of EBF was the greatest during the first two months of life, across all countries. Afghanistan had the lowest number (39.3%) of babies who were EBF relative to Bangladesh, India, and Nepal where more than 50% of babies were EBF. With respect to initiation of breastfeeding, only one fifth (20.7%) of the babies in Pakistan were breastfed within the first hour of birth, the lowest out of 5 South Asian countries. Furthermore, Pakistan also had the highest proportion (38.6%) of babies who were bottle fed. Pakistan also had the highest proportion of babies experiencing diarrhoeal episodes (22.8%), ARI (13.8%), and fever (35.1%) in the 2 weeks before the survey, compared to the other countries.

### Prevalence of morbidity outcomes by EBF

**Fig. 2:**
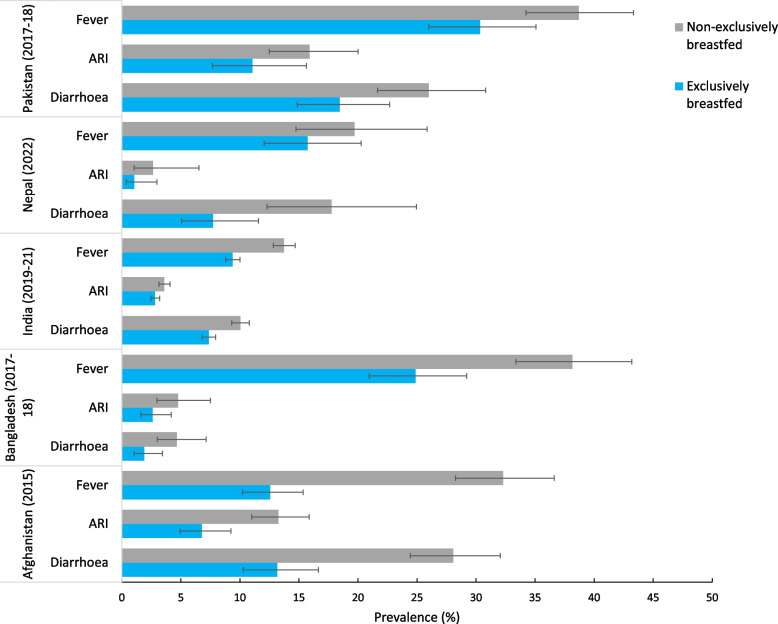
Prevalence of babies aged 0–6 months with morbidity outcomes (diarrhoea, acute respiratory infections, and fever) in the 2 weeks preceding the survey by exclusive breastfeeding status in South Asian countries. Error bars represent 95% confidence intervals (CI). ARI: Acute respiratory infection

In India, the prevalence of diarrhoea in the 2 weeks before the survey was lower in babies who were EBF (7.4%) compared to babies who were not EBF (10%) (See Fig. 2). Similarly, the prevalence of fever was lower in babies who were EBF (9.4%) compared to babies who were not EBF (13.7%). There was no difference in ARI prevalence among babies who were EBF and those who were not EBF in India. In Bangladesh, the prevalence of fever was lower in babies who were EBF (24.9%) compared to babies who were not EBF (38.2%). However, there was no difference in diarrhoea and ARI prevalence among babies who were EBF and those who were not EBF. In Afghanistan, diarrhoea (13.1% vs. 28.1%), ARI (6.8% vs. 13.2%), and fever (12.6% vs. 32.3%) were less prevalent in babies who were EBF compared to those were not. Lower prevalence estimates of diarrhoea were also observed in infants in Nepal who were EBF (7.7%), compared to those who were not EBF (17.8%). In Pakistan, there was no significant difference in diarrhoea, ARI, and fever prevalence among babies who were EBF and those who were not EBF (See Fig. 2).

### Association of EBF and morbidity outcomes

**Fig. 3:**
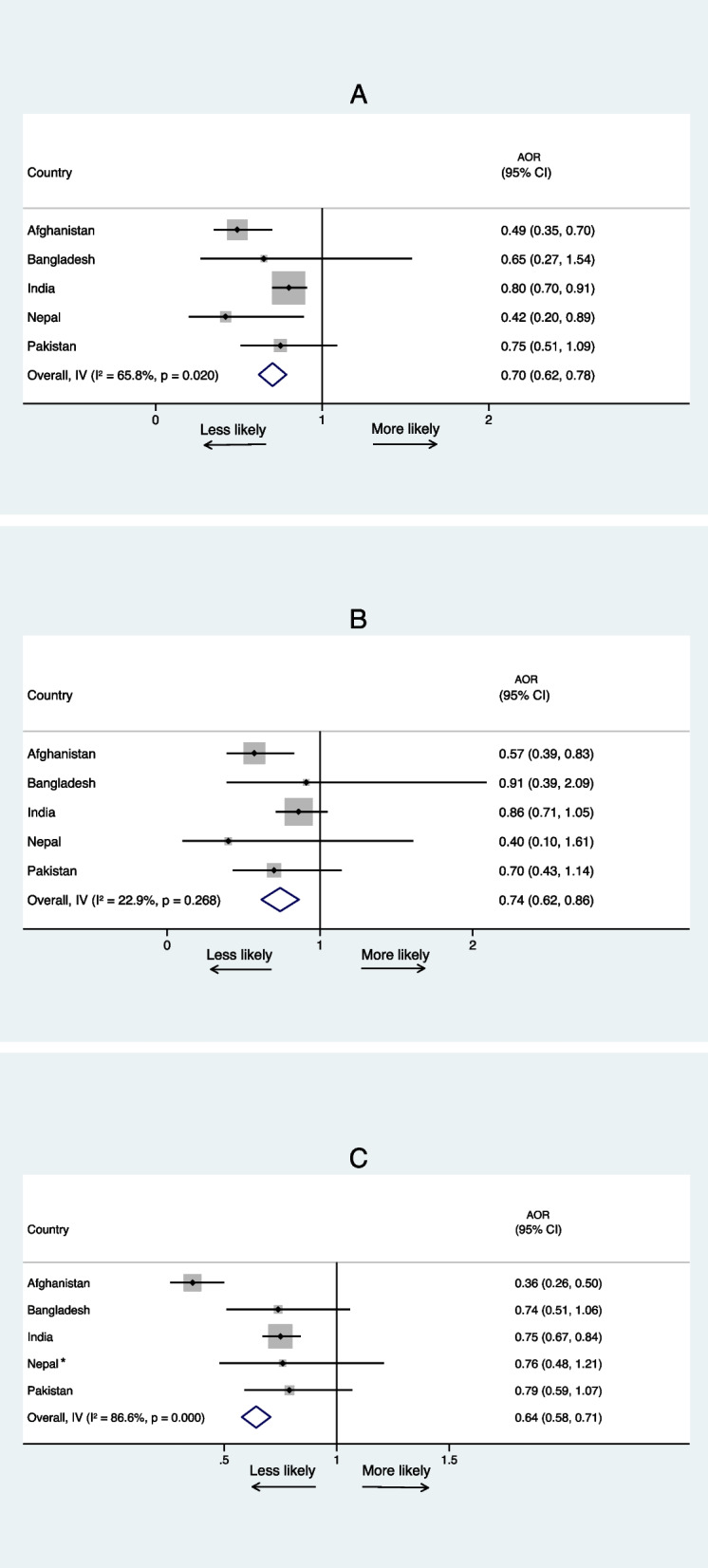
Summary of the association between exclusive breastfeeding (EBF) and infant morbidity outcomes in 5 South Asian countries. **A** Association between EBF and diarrhoea. **B** Association between EBF and acute respiratory infection. **C** Association between EBF and fever. Estimates by country weighted according to study design; overall estimates were not weighted. * Unadjusted odd ratios for EBF and ARI and fever in Nepal in **B**, **C**. AOR: Adjusted Odds Ratio; CI: Confidence interval

In the multivariable analysis, EBF was associated with decreased odds of having diarrhoeal disease in the 2 weeks before the survey among babies aged 0–6 months in India (AOR: 0.80, 95% CI 0.70, 0.91), Afghanistan (AOR: 0.49, 95% CI 0.35, 0.70), and Nepal (AOR: 0.42, 95% CI 0.20, 0.89) compared to those who were not EBF (Table 3). Infants in Afghanistan who were EBF were also less likely to experience ARI compared to those who were not EBF (AOR: 0.57, 95% CI 0.39, 0.83) (Table 3). EBF was associated with lower odds of fever among Indian (AOR: 0.75, 95% CI 0.67, 0.84) and Afghani infants (AOR: 0.36, 95% CI 0.26, 0.50) (Table 4) Similar direction of effects are also observed in Pakistan and Bangladesh, however the results are non-significant. These results are illustrated in Fig. 3.

**Table 3:** Association of exclusive breastfeeding and various individual, household-level, and socio-demographic factors with diarrhea and acute respiratory infection^a^ in South Asia

	**Diarrhoea in the last 2 weeks**					**Acute respiratory infection in the last 2 weeks**				
	**Afghanistan (2015)** (*n* = 3462)	**Bangladesh (2017/18)** (*n* = 1084)	**India (2019/21)** (*n* = 26101)	**Nepal (2022)** (*n* = 581)	**Pakistan (2017/18)** (*n* = 1306)	**Afghanistan (2015)**^b^ (*n* = 3467)	**Bangladesh (2017/18)** (*n* = 1084)	**India (2019/21)** (*n* = 26101)	**Nepal (2022)**^c^ (*n* = 581)	**Pakistan (2017/18)** (*n* = 1306)
**Variables**	**AOR (95% CI)**	**AOR (95% CI)**	**AOR (95% CI)**	**AOR (95% CI)**	**AOR (95% CI)**	**AOR (95% CI)**	**AOR (95% CI)**	**AOR (95% CI)**	**AOR (95% CI)**	**AOR (95% CI)**
**Infant age (months)**										
0–2	Ref	Ref	Ref	Ref	Ref	Ref	Ref	Ref		Ref
3–4	2.54 (1.70, 3.79)**	3.55 (1.05, 12.05)*	1.61 (1.37, 1.88)**	1.30 (0.65, 2.61)	1.19 (0.77, 1.85)	1.69 (1.02, 2.83)*	2.11 (0.78, 5.68)	1.49 (1.18, 1.89)*		2.12 (1.25, 3.59)*
5–6	2.95 (2.11, 4.11)**	4.02 (1.13, 14.32)*	1.81 (1.55, 2.13)**	1.56 (0.69, 3.56)	1.80 (1.10, 2.93)*	2.18 (1.34, 3.56)*	3.90 (1.39, 10.91)*	1.69 (1.32, 2.15)**		2.46 (1.41, 4.28)*
**Gender**										
Male			Ref				Ref			
Female			0.86 (0.76, 0.97)*				0.43 (0.20, 0.94)*			
**Mother’s age in years**										
15–19			Ref		Ref	Ref		Ref		
20–29			0.75 (0.61, 0.93)*		0.78 (0.45, 1.35)	1.83 (0.90, 3.74)		0.80 (0.56, 1.15)		
30–39			0.54 (0.42, 0.70)**		0.50 (0.26, 0.98)*	1.85 (0.86, 3.99)		0.71 (0.46, 1.08)		
40–49			0.53 (0.26, 1.03)		1.35 (0.52, 3.55)	1.95 (0.74, 5.19)		0.27 (0.09, 0.84)*		
**Maternal education**										
No education	Ref	Ref		Ref	Ref			Ref		Ref
Primary	1.07 (0.60, 1.91)	0.29 (0.08, 1.11)		1.12 (0.49, 2.58)	2.36 (1.43, 3.91)*			1.00 (0.73, 1.37)		1.18 (0.63, 2.19)
Secondary	1.36 (0.87, 2.14)	0.30 (0.09, 1.02)		1.05 (0.42, 2.63)	1.51 (0.87, 2.59)			1.02 (0.79, 1.31)		1.52 (0.81, 2.83)
Higher	0.66 (0.27, 1.63)	0.23 (0.05, 0.99)*		0.12 (0.01, 1.15)	1.57 (0.75, 3.26)			0.84 (0.59, 1.20)		0.35 (0.14, 0.86)*
**Mother’s employment**										
Not working										Ref
Working										1.29 (0.60, 2.80)
**Type of residence**										
Urban	Ref		Ref							
Rural	0.86 (0.54, 1.36)		1.20 (0.99, 1.45)							
**Household size (members)**										
1–4	Ref			Ref						
5–8	0.57 (0.30, 1.08)			2.21 (0.94, 5.16)						
9 +	0.51 (0.30, 0.87)*			3.51 (1.27, 9.67)*						
**Wealth index**										
Poorest			Ref		Ref		Ref	Ref		Ref
Poorer			1.25 (1.05, 1.49)*		1.45 (0.89, 2.35)		0.81 (0.32, 2.03)	0.97 (0.75, 1.25)		1.07 (0.57, 2.03)
Middle			1.09 (0.90, 1.31)		1.01 (0.52, 1.93)		0.59 (0.19, 1.85)	1.00 (0.75, 1.33)		0.62 (0.26, 1.49)
Richer			1.07 (0.88, 1.32)		1.33 (0.72, 2.43)		0.59 (0.20, 1.75)	0.79 (0.58, 1.07)		0.96 (0.38, 2.44)
Richest			0.87 (0.66, 1.15)		0.61 (0.29, 1.30)		0.32 (0.10, 0.95)*	0.64 (0.45, 0.91)*		1.01 (0.36, 2.83)
**Source of drinking water**										
Improved	Ref					Ref				
Unimproved	0.86 (0.63, 1.18)					1.36 (0.95, 1.94)				
**Type of toilet facility**										
Improved	Ref		Ref							Ref
Unimproved	0.83 (0.59, 1.17)		1.08 (0.93, 1.25)							1.35 (0.72, 2.55)
**Type of cooking fuel**										
Clean										
Solid										
**Number of ANC visits**										
None	Ref					Ref		Ref		
1–3	1.40 (0.96, 2.02)					2.01 (1.13, 3.56)*		2.10 (1.31, 3.37)*		
4 or more	1.96 (1.31, 2.93)*					2.42 (1.21, 4.83)*		1.90 (1.19, 3.04)*		
**Place of delivery**										
Home						Ref				
Health facility						0.48 (0.31, 0.75)*				
**Exclusive breastfeeding**										
No	Ref	Ref	Ref	Ref	Ref	Ref	Ref	Ref		Ref
Yes	0.49 (0.35, 0.70)**	0.65 (0.27, 1.54)	0.80 (0.70, 0.91)*	0.42 (0.20, 0.89)*	0.75 (0.51, 1.09)	0.57 (0.39, 0.83)*	0.91 (0.39, 2.09)	0.86 (0.71, 1.05)		0.70 (0.43, 1.14)

Only variables which met the cutoff for significance (*p* < 0.10) were included in the multivariable model

*AOR* adjusted odds ratio, *Ref* Reference category

Level of significance: *p < 0.05, **p < 0.001

^a^Diarrhoea and acute respiratory infection symptoms in the previous 2 weeks

^b^Sample sizes different in table for Afghanistan DHS as models differed by outcomes

^c^Unadjusted odd ratios for EBF in Nepal not significant

**Table 4:** Association of exclusive breastfeeding and various individual, household-level, and socio-demographic factors with fever^a^ in South Asia

	**Fever in the last 2 weeks**				
	**Afghanistan (2015)**^b^ (*n* = 3460)	**Bangladesh (2017/18)** (*n* = 1084)	**India (2019/21)** (*n* = 26101)	**Nepal (2022)** (*n* = 581)	**Pakistan (2017/18)** (*n* = 1306)
**Variables**	**AOR (95% CI)**	**AOR (95% CI)**	**AOR (95% CI)**	**AOR (95% CI)**	**AOR (95% CI)**
**Infant age (months)**					
0–2	Ref	Ref	Ref		Ref
3–4	1.52 (1.02, 2.26)*	1.60 (1.09, 2.34)*	1.84 (1.60, 2.11)**		1.56 (1.09, 2.22)*
5–6	1.83 (1.24, 2.71)*	2.11 (1.40, 3.19)**	1.95 (1.69, 2.25)**		2.29 (1.57, 3.34)**
**Gender**					
Male		Ref	Ref		
Female		0.75 (0.56, 0.99)*	0.90 (0.81, 1.01)		
**Mother’s age in years**					
15–19			Ref		Ref
20–29			0.67 (0.55, 0.82)**		0.67 (0.38, 1.18)
30–39			0.58 (0.45, 0.74)**		0.63 (0.35, 1.13)
40–49			0.43 (0.24, 0.80)*		0.44 (0.16, 1.19)
**Maternal education**					
No education			Ref		Ref
Primary			1.13 (0.93, 1.38)		1.84 (1.13, 3.01)*
Secondary			1.07 (0.91, 1.24)		2.30 (1.47, 3.61)**
Higher			0.86 (0.69, 1.08)		0.88 (0.46, 1.68)
**Mother’s employment**					
Not working		Ref			Ref
Working		1.33 (0.98, 1.81)			2.02 (1.14, 3.61)*
**Type of residence**					
Urban			Ref		
Rural			0.92 (0.77, 1.10)		
**Household size (members)**					
1–4					
5–8					
9 +					
**Wealth index**					
Poorest			Ref		Ref
Poorer			0.87 (0.74, 1.03)		1.11 (0.65, 1.92)
Middle			0.87 (0.72, 1.05)		0.72 (0.43, 1.22)
Richer			0.65 (0.53, 0.81)**		0.87 (0.49, 1.55)
Richest			0.53 (0.41, 0.70)**		0.52 (0.27, 1.01)
**Source of drinking water**					
Improved					
Unimproved					
**Type of toilet facility**					
Improved	Ref		Ref		
Unimproved	0.83 (0.58, 1.20)		1.18 (1.05, 1.33)*		
**Number of ANC visits**					
None	Ref		Ref		
1–3	1.51 (1.09, 2.08)*		0.85 (0.68, 1.08)		
4 or more	1.99 (1.28, 3.08)*		0.73 (0.58, 0.92)*		
**Place of delivery**					
Home			Ref		
Health facility			0.84 (0.71, 1.00)		
**Exclusive breastfeeding**					
No	Ref	Ref	Ref		Ref
Yes	0.36 (0.26, 0.50)**	0.74 (0.51, 1.06)	0.75 (0.67, 0.84)**		0.79 (0.59, 1.07)

Only variables which met the cutoff for significance (*p* < 0.10) were included in the multivariable model

*AOR* adjusted odds ratio, *Ref* Reference category

Level of significance: *p < 0.05, **p < 0.001

^a^Fever symptoms in the previous 2 weeks

^b^Sample size different between tables for Afghanistan DHS as models differed by outcomes

### Association of other IYCF indicators and morbidity outcomes

In India, babies who received breastfed within the first hour of birth had lower odds of experiencing diarrhoea in the 2 weeks before the survey compared to those who did not receive breastfed within the first hour of birth (AOR: 0.79, 95% CI 0.69, 0.90) (Table 5). We also found that infants who were bottle fed were 1.48 times more likely to experience diarrhoea in India (AOR: 1.48, 95% CI 1.26, 1.75) compared to their counterparts (Table 5). Similarly, babies who were bottle fed were more likely to have ARI in the 2 weeks before the survey relative to those who were not bottle fed in India (AOR: 1.38, 95% CI 1.09, 1.75) and Afghanistan (AOR: 2.53, 95% CI 1.57, 3.53) (Table 5). Infants who were bottle fed were 1.37 and 2.09 times more likely to have fever in India (AOR: 1.37, 95% CI 1.17, 1.59) and Afghanistan (AOR: 2.09, 95% CI 1.49, 2.91) compared to their counterparts (Table 6).

**Table 5:** Adjusted logistic regression models for association between other IYCF indicators and diarrhoea and acute respiratory infections in 5 South Asian countries

	**Diarrhoea in the last two weeks**^a^					**Acute respiratory infection in the last two weeks**^b^				
***IYCF indicators***	**Afghanistan (2015)** (*n* = 3462) **AOR (95% CI)**	**Bangladesh (2017/18)** (*n* = 1084) **AOR (95% CI)**	**India (2019/21)** (*n* = 26101) **AOR (95% CI)**	**Nepal (2022)** (*n* = 581) **AOR (95% CI)**	**Pakistan (2017/18)** (*n* = 1306) **AOR (95% CI)**	**Afghanistan (2015)** (*n* = 3462) **AOR (95% CI)**	**Bangladesh (2017/18)** (*n* = 1084) **AOR (95% CI)**	**India (2019/21)** (*n* = 26101) **AOR (95% CI)**	**Nepal (2022)** (*n* = 581) **AOR (95% CI)**	**Pakistan (2017/18)** (*n* = 1306) **AOR (95% CI)**
**Early initiation of breastfeeding**										
No	-	-	Ref	-	Ref	-	-	Ref	Ref	-
Yes	-	-	0.79 (0.69, 0.90)**	-	1.40 (0.94, 2.06)	-	-	0.83 (0.68, 1.01)	4.21 (0.82, 21.78)	-
**Bottle feeding**										
No	Ref	-	Ref	-	-	Ref	-	Ref	-	-
Yes	1.36 (0.98, 1.88)	-	1.48 (1.26, 1.75)**	-	-	2.35 (1.57, 3.53)**	-	1.38 (1.09, 1.75)*	-	-

*Ref* Reference category

Level of significance: *p < 0.05, **p < 0.001

^a^ORs were adjusted for infant age, gender, mother’s age, maternal education, mother’s employment, type of residence, household size, wealth index, source of drinking water, toilet facility, number of ANC visits, place of delivery

^b^ORs were adjusted for infant age, gender, mother’s age, maternal education, mother’s employment, type of residence, household size, wealth index, source of drinking water, toilet facility, number of ANC visits, place of delivery, and cooking fuel

- not included in multivariate analysis as *p* value in bivariable analysis not significant

**Table 6:** Adjusted logistic regression model for association between other IYCF indicators and fever in 5 South Asian countries

	**Fever in the last 2 weeks**				
***IYCF indicators***	**Afghanistan (2015)** (*n* = 3460) **AOR (95% CI)**	**Bangladesh (2017/18)** (*n* = 1084) **AOR (95% CI)**	**India (2019/21)** (*n* = 26101) **AOR (95% CI)**	**Nepal (2022)** (*n* = 581) **AOR (95% CI)**	**Pakistan (2017/18)** (*n* = 1306) **AOR (95% CI)**
**Early initiation of breastfeeding**					
No	-	-	-	-	-
Yes	-	-	-	-	-
**Bottle feeding**					
No	Ref	Ref	Ref	-	-
Yes	2.09 (1.49, 2.91)**	1.34 (0.89, 2.01)	1.37 (1.17, 1.59)**	-	-

ORs were adjusted for infant age, gender, mother’s age, maternal education, mother’s employment, type of residence, household size, wealth index, source of drinking water, toilet facility, number of ANC visits, and place of delivery

*Ref* Reference category

Level of significance: **p < 0.001

- not included in multivariate analysis as *p* value in bivariable analysis not significant

## Discussion

### Main findings

Based on our analysis of nationally representative datasets in five South Asian countries, EBF is a protective factor for diarrhoea in infants aged 0–6 months in Afghanistan, India, and Nepal and for fever in Afghanistan and India. Our study also showed that infants who were EBF had lower odds of ARI in Afghanistan. These findings are consistent with data from other studies in LMICs which also estimated the beneficial association of EBF [29, 33–36, 40]. A large study [34] that included data from the 2015–2016 India DHS found that EBF was protective against diarrhoea in babies aged 0 to 5 months at a national level (AOR: 0.64, 95% CI 0.57, 0.72) as well as in the Central, Northern and Eastern regions of the country. Another large study [29] that pooled data from the DHS in nine sub-Saharan African countries with high rates of diarrhoeal morbidity reported that EBF was significantly associated with decreased likelihood of diarrhoea among babies aged 0–5 months (AOR: 0.50, 95% CI 0.43, 0.57). A cross-sectional study in Vietnam [41] suggested that babies who were predominantly or partially breastfed had a greater likelihood of having diarrhoea in comparison to babies who were EBF. Individual studies in Bangladesh [33, 35] and Pakistan [36] revealed that EBF reduces the risk of diarrhoea, ARI, and fever. Although our study found a protective effect of EBF in Bangladesh and Pakistan where the general direction of the results was similar with Afghanistan, India, and Nepal, these results were non-significant. This inconsistency in findings could be attributed to the considerable smaller sample size of the surveys in these two countries compared to Afghanistan and India.

Several biological mechanisms explain why EBF may have protective effects on infectious diseases such as diarrhoea and ARI. Breast milk contains numerous anti-inflammatory, antimicrobial, growth factors and bioactive elements such as oligosaccharides, immunoglobulin A (IgA), and lactoferrin that protect against childhood infections [4, 5, 7]. A baby’s immunological development and maturation are potentially aided by human milk, which has its own immune system and a variety of soluble and cellular components [7]. IgA stops bacteria and viruses from adhering to the mucosal epithelial cells, which might lead to infections [4, 5, 7]. Additionally, there is a theory that oligosaccharides may prevent respiratory infections and gastrointestinal illnesses in babies by preventing pathogens from attaching to their mucosa [4, 5, 7]. The primary protein in human milk is lactoferrin, which functions as a microbicidal agent to eradicate viruses and bacteria [4, 5, 7]. Furthermore, infant weaning foods and powdered infant formula have been shown to be contaminated with pathogens, putting non-breastfed newborns at an increased risk of exposure to these contaminants [42–44]. Powdered infant formula is not sterile and has been associated with significant illness from infections with the bacteria *Cronobacter sakazakii* and *Salmonella* spp [43, 45]. For example, a study in Nigeria found that *Cronobacter sakazakii* was found in 16 of the 360 powdered infant formula samples that were examined, representing an average prevalence rate of 4.4% [42]. Also, other sources of contamination during formula milk preparation include the addition of unclean water, improper handling or inadequate nipple and bottle cleaning [45].

Given its potential to have a significant negative impact on health outcomes, prevalence of bottle feeding is a IYCF indicators [32]. Our study found that babies who were bottle fed were more likely to have diarrhoea in India, ARI in Afghanistan, and fever in India and Afghanistan. The similar pattern across these South Asian countries suggests a high degree of consistency in the association between bottle feeding and diarrhoea, ARI, and fever. This pattern was also observed in earlier investigations in India [34] and Ethiopia [46] which have reported the impact of bottle feeding. The recent paper in Ethiopia [46] revealed that babies and young children aged 0–23 months who receiving bottle feeding were 1.36 times more likely to experience ARI relative to those who did not receive bottle feeding. Also, children who were bottle fed tested positive for rotavirus infection, the most prevalent cause of diarrhoea, according to an observational study [47] done in a hospital in India. This may be due to the higher risk of contamination from the water used to make breastmilk substitute, bottle, teat, or milk in babies who are fed bottle milk [5]. In Peru, an investigation [48] showed that 23% of bottles and 35% of bottle nipples were contaminated with *Escherichia coli*, which was greater than any other household item tested.

Another key IYCF indicator is early initiation of breastfeeding, within an hour of birth. Our analysis indicated that early initiation of breastfeeding was associated with lower diarrhoeal disease in India. This result was consistent with a past study conducted on the DHS data in India [34] and sub-Saharan African nations [29].

Around 27% of all deaths in children under five occurred in the South Asian region according to UNICEF in 2020 [14]. A meta-analysis [49] undertaken estimated that babies who were predominantly breastfed, partially breastfed, and non-breastfed had a greater risk of death (relative risk of 1.48, 2.84, and 14.4 respectively) relative to babies 0–5 months of age who were EBF respectively in low-income countries. One of the most cost-effective child survival interventions is EBF during the first six months of life, which significantly lowers the chances of a child dying from pneumonia or diarrhoea [6, 16]. Breastfeeding promotes development gains at all levels, from decreased illness incidence to economic returns, and will be a significant factor in reaching the Sustainable Development Goals (SDG) 2 and 3 on avoiding child deaths, attaining food security, and improving nutrition in South Asia [50]. To successfully establish a more supportive environment for mothers who choose to breastfeed, we require investments from governments at all levels of society [4]. This involves providing women with the information they need to make informed choices, as well as the support they require from their families, communities, work environments, and healthcare systems, in order to ensure that EBF for the first six months is possible [4]. Increased protection, promotion, and support for EBF would offer a cost-effective approach towards reaching the SDGs.

In South Asia, various factors have an influence on EBF. For instance, compared to boys, girls are less likely to EBF in India [19]. Moreover, in contrast to some low caste groups in India, the Tajik and other smaller four ethnic groups in Afghanistan are less likely to EBF, which implies that sociocultural norms may have an impact on EBF and are context specific [19]. A qualitative study in Pakistan in 2018 revealed that negative attitudes towards colostrum, poor social support, influence of social and family decision-makers, perceived inadequate milk supply, mother’s heavy workload, and advertising of infant formula were all factors that were barriers to optimal EBF practices [51]. Therefore, these highlight the need for context-specific and integrated interventions tailored to each South Asian country to support mothers in overcoming the barriers to optimal breastfeeding practices. The promotion of laws and rules governing the marketing of breast milk substitutes, supporting breastfeeding in the workplace and households and paid maternity leave in LMICs is crucial [28].

### Strengths and limitations

Potential limitations of the study should be taken into consideration when interpreting the results. The statistical findings revealed significant effects for Afghanistan and India, which are, respectively, large sample populations, and highly deprived environments where the impacts of EBF are large. It is likely that the absence of significant results for the three nations with smaller samples (Bangladesh, Nepal, and Pakistan) may be due to a lack of statistical power, or Type II error. A paper published in 1994 showed the extent of underpowered studies that led to null trials in the literature [52]. Only 36% of the null trials included in the survey had sufficient power (80%) to identify a relative difference of 50% [52]. The “absence of evidence” in these studies should not be regarded as “evidence of absence,” and underpowered studies should be interpreted with caution [53].

Given that the data were gathered via self-reports, recall bias may have had an impact on the findings. Nevertheless, we restricted the study sample to the youngest living baby who resided with the mother to lessen the possible impact of recall bias. Also, our study used cross-sectional data, which makes it challenging to establish a causal relationship. Another important limitation is that there may have been potential misclassification bias, as measurement of ARI, fever, and diarrhoea was determined in the two weeks before the survey. This implies that mothers could have inaccurately stated that their babies had symptoms of ARI, fever, or diarrhoea resulting in an underestimation or overestimation of the measure of association between EBF and other IYCF indicators and morbidity outcomes. Another limitation is that we were not able to adjust for all the confounding variables such as seasonal and cultural variations which could have affected the association between the exposure and outcomes. An important limitation to consider is that dichotomous feeding exposures in nations where breastfeeding is practiced differently, EBF and not EBF have varied meanings; because of this, when comparing EBF and not EBF in nations such as Bangladesh, India, and Nepal with high rates of both exclusive and partial breastfeeding, the magnitude of the benefits of EBF relative to commercial milk formula or bottle feeding is understated. For nations like Nepal with a relatively small population size in the surveys, there may be very few bottle-fed babies in comparisons with the non-EBF group, which could provide challenges for the statistical analysis. Additionally, for similar reasons, cross-country comparisons of EBF and not EBF results may be inaccurate for nations like Pakistan that have relatively low breastfeeding rates.

A key strength is that our study uses the latest data from nationally representative sample from each South Asian country included to enable sufficient generalizability of study findings. Finally, due to the high response rate in the surveys and the use of consistent standardized questionnaires, the study results are less likely to be impacted by selection bias.

## Conclusion

In summary, our study has shown that babies aged 0–6 months who were EBF had lower odds of diarrhoea in Afghanistan, India, and Nepal. EBF was also associated with lower odds of fever in Afghanistan and India. Additionally, EBF was a protective factor against ARI in Afghanistan. Bottle feeding was a risk factor for diarrhoea and ARI in some South Asian countries and early initiation of breastfeeding was protective against diarrhoea in India. The results of this study seem to suggest that EBF, if expanded to universal levels may have an impact on morbidity in some South Asian countries. These findings strengthen the evidence base showing that EBF is a critical intervention to reduce morbidity and further advocacy efforts should be used to promote, support and improve breastfeeding practices in South Asia.

### Supplementary Information

Additional file 1: Table S1. Unadjusted logistic regression results for association of exclusive breastfeeding and various individual, household-level, and socio-demographic factors with diarrhea, acute respiratory infection, and fever among infants 0-6 months in South Asia. Table S2. Unadjusted logistic regression results for association of other IYCF indicators and various individual, household-level, and socio-demographic factors with diarrhea, acute respiratory infection, and fever among infants 0-6 months in South Asia.

## Data Availability

Datasets used for this paper are available online at: https://dhsprogram.com/data/available-datasets.cfm.

## Abbreviations

ALRI: Acute Lower Respiratory Infections
ANC: Antenatal Care
AOR: Adjusted Odds Ratios
ARI: Acute Respiratory Infection
CI: Confidence interval
DHS: Demographic and Health Surveys
EBF: Exclusive Breastfeeding
IgA: Immunoglobulin A
IYCF: Infant and Young Child Feeding
JMP: Joint Monitoring Programme
LMIC: Low-and Middle-Income Country
SDG: Sustainable Development Goal
UNICEF: United Nations Children’s Fund
VIF: Variance Inflation Factor
VIP: Ventilated Improved Pit
WHO: World Health Organization
